# Retrospective analysis of 1539 nasopharyngeal carcinoma cases: chemotherapy should not be excluded for non-Asian patients with T1-2N1M0 stage

**DOI:** 10.3389/fonc.2024.1529136

**Published:** 2025-01-17

**Authors:** Xin-Yu Li, Chang-Ying Zhong, Hui-Xian Xu

**Affiliations:** ^1^ Institute of Rehabilitation and Health Care, Department of Rehabilitation and Traditional Chinese Medicine, Hunan Traditional Chinese Medical College, Zhuzhou, Hunan, China; ^2^ Otolaryngology Department, Hunan Provincial Hospital of Integrated Traditional Chinese and Western Medicine, Changsha, Hunan, China; ^3^ Department of Otolaryngology Head and Neck Surgery, The First Affiliated Hospital of Guangzhou University of Chinese Medicine, Guangzhou, Guangdong, China

**Keywords:** nasopharyngeal carcinoma, chemotherapy, Asian, stage II, proportional hazard model

## Abstract

**Background:**

Many results suggested that chemotherapy cannot provide survival benefit for stage II nasopharyngeal carcinoma. It remained unclear whether the efficacy of chemotherapy differed in non-Asian populations.

**Objective:**

It was designed to analyze the effect of chemotherapy for Asian and non-Asian patients with stage II nasopharyngeal carcinoma.

**Method:**

Patients were collected using the SEER program. The variables included age, sex, race, marital status, survival time, survival status, TNM stage, radiation and chemotherapy. Utilizing the Rstudio (version: 2024.4.1.748) and R (version: 4.4.1), backward elimination method was employed to screen the variables and multivariate Cox regression analyses was conducted on the screened variables. Kaplan-Meier method was utilized to analyze the survival of sub-stages and different races with T1-2N1M0 stage. The log-rank test was used for statistical analysis.

**Result:**

1539 patients were collected. Chemotherapy was statistically significant, with a hazard ratio (HR) of 0.64, P=0.003 in stage II patients. The HR for radiation was 0.33, P<0.001. Chemotherapy didn’t improve cancer-specific survival for patients with T2N0M0 stage. Asian and non-Asian races showed no difference in cancer-specific survival in T2N0M0 stage with HR of 1.85, P=0.13. For patients with T1-2N1M0 stage, chemotherapy improved cancer-specific survival with a HR of 0.53, P<0.001. No significant difference was in the Kaplan-Meier analysis between the two sub-stages (P=0.065). In T1-2N1M0 stage, multivariate Cox regression analysis for Asian race indicated that chemotherapy didn’t improve cancer-specific survival with a HR of 0.64, P=0.190. For non-Asian race, chemotherapy was found to improve cancer-specific survival, with a HR of 0.51, P<0.001. The Kaplan-Meier analysis of Asian and non-Asian patients with T1-2N1M0 stage exhibited significant differences (P<0.0001).

**Conclusion:**

Chemotherapy is correlated with the cancer-specific survival in non-Asian patients with T1-2N1M0-stage nasopharyngeal carcinoma, but not in Asian patients at the same stage. For patients with the T2N0M0 stage, chemotherapy is not correlated with the cancer-specific survival rate, regardless of ethnicity.

## 1 Introduction

Nasopharyngeal carcinoma (NPC) is a rare malignancy arising from the nasopharyngeal epithelium with global incidence varying by region. It was more prevalent in China and Southeast Asian countries (1). Within China, the incidence was higher in the southern regions compared to the northern ones (2). In 2018, 129,000 people were diagnosed with NPC worldwide, a number that rose to 133,000 in 2020 (3). 60,000 cases were diagnosed in China among the 133,000 patients, which accounts for 46.8% (4). Although NPC is considered rare on a global scale, it remains a significant health concern and a research focus in China and other southeast Asian countries due to its higher incidence rates. In the recent review, a novel and interesting view was that nasopharyngeal carcinoma should not be defined as a disease caused by genetics or genetic mutations, but as a pathological ecosystem, which means gene mutations is one of causes (5). This view attempted to integrate ecological concepts into pathology and oncology and tries to integrate them into a new discipline: Integrated tumoriecology.

NPC is categorized into four clinical stages (I, II, III, IV), each with distinct treatment options. A point of contention arose in the treatment of stage II, where there was disagreement on the necessity of incorporating chemotherapy for patients at this stage or its sub-stages. Stage II NPC encompasses two sub-stages: one with lymph node metastasis (T1-2N1M0) and the other without lymph node metastasis (T2N0M0). The Chinese Society of Clinical Oncology (CSCO) and the European Society for Medical Oncology (ESMO) offered divergent suggestions. CSCO acknowledged that existing studies were insufficient to confirm the efficacy of chemotherapy for stage II patients and concurrent chemoradiotherapy (CCRT) was recommended for T1-2N1M0 stage and intensity-modulated radiation therapy (IMRT) for T2N0M0 stage (6). ESMO recommended IMRT alone for stage II (7).

Moreover, the majority of patients in NPC trials hailed from China or other Asian countries, with most results being based on the Asian population. It remained unclear whether chemotherapy was effective for non-Asian patients at stage II, as previous findings have indicated that Asians might have a cancer-specific survival (CSS) advantage compared to other races (8).

The objective of this study was to assess the impact of chemotherapy on CSS for patients with stage II NPC, including its sub-stages, and to determine if there were differences in treatment effect of chemotherapy between Asian and non-Asian populations.

## 2 Materials and methods

### 2.1 Date source and cases inclusion

The Surveillance, Epidemiology, and End Results (SEER) program, established by the National Cancer Institute (NCI), encompassed a comprehensive dataset of cancer incidence, overall survival, cancer-specific survival, and treatment information in the United States. We have selected data from 17 registries spanning from the years 2000 to 2021 for our analysis.

For a thorough data collection, we applied the following inclusion criteria: (1) patients diagnosed between 2004 and 2021; (2) tumors classified according to the ICD-O-3/WHO 2008 system, with a focus on ‘nasopharynx’ as the primary site; (3) age ranged from 1 to 89 years; (4) both genders were included; (5) TNM stage was restricted to T1-2N1M0 and T2N0M0 after screening.

### 2.2 The selection of variables

The selection of variables was described as follows: (1)age at diagnosis, (2) race, (3) sex, (4) marital status, (5) SEER cause-specific death classification, and survival time (months), (6) radiation, (7) chemotherapy.

### 2.3 Merging and exclusion of data

Since the included cases spanned from 2004 to 2021, patients were classified using different editions of the TNM stage classification. The 6th edition was too restrictive to include cases at T1 stage, while the 8th edition was not suitable for patients between 2004 and 2017. Therefore, we adopted the 7th edition of the TNM classification as our criterion. This decision was based on the fact that the 6th edition’s criteria could be integrated into the 7th. 8th can optimize 7th. The merging rules were detailed in **Table 1**. The code in **Table 1** were from SEER Cancer Schema list of nasopharynx.

**Table 1:** Principle of T and N classification.

AJCC 6th	Data merging code (AJCC 6th to AJCC 7th)	AJCC 7th	Code Description
T2NOS	505^a^	T2	Extension to soft tissue (excluding soft tissue of neck)
T1	105, 205, 305	T1	Involvement of two or more subsites
T2a	400, 500	T1	Without parapharyngeal extension
T2b	555, 560, 565, 590	T2	Parapharyngeal extension or defined by SEER database
T2b	580^b^, 585^b^	T3 (excluded)	Pterygopalatine fossa
N1	500, 505, 515, 520	Unknown (excluded)	Unknown bilateral or unilateral of neck
N1	620	N3 (excluded)	Lymph node size over 6 cm
N1	50, 60, 70	N1	Unilateral or bilateral positive lymph node (s): retropharyngeal
N1	100,105,110,115, 20,125,130,180, 200,210,220,300, 310,320,800	N1	Unilateral positive regional node (s) of neck over supraclavicular fossa

^a^Cases with code “505” would be transformed T1 stage into T2 stage due to its severity.

^b^Cases with code “580” and “585” would be transformed from T2 stage into T3 stage due to its severity.

### 2.4 Multivariate regression analysis

**Figure 1:**
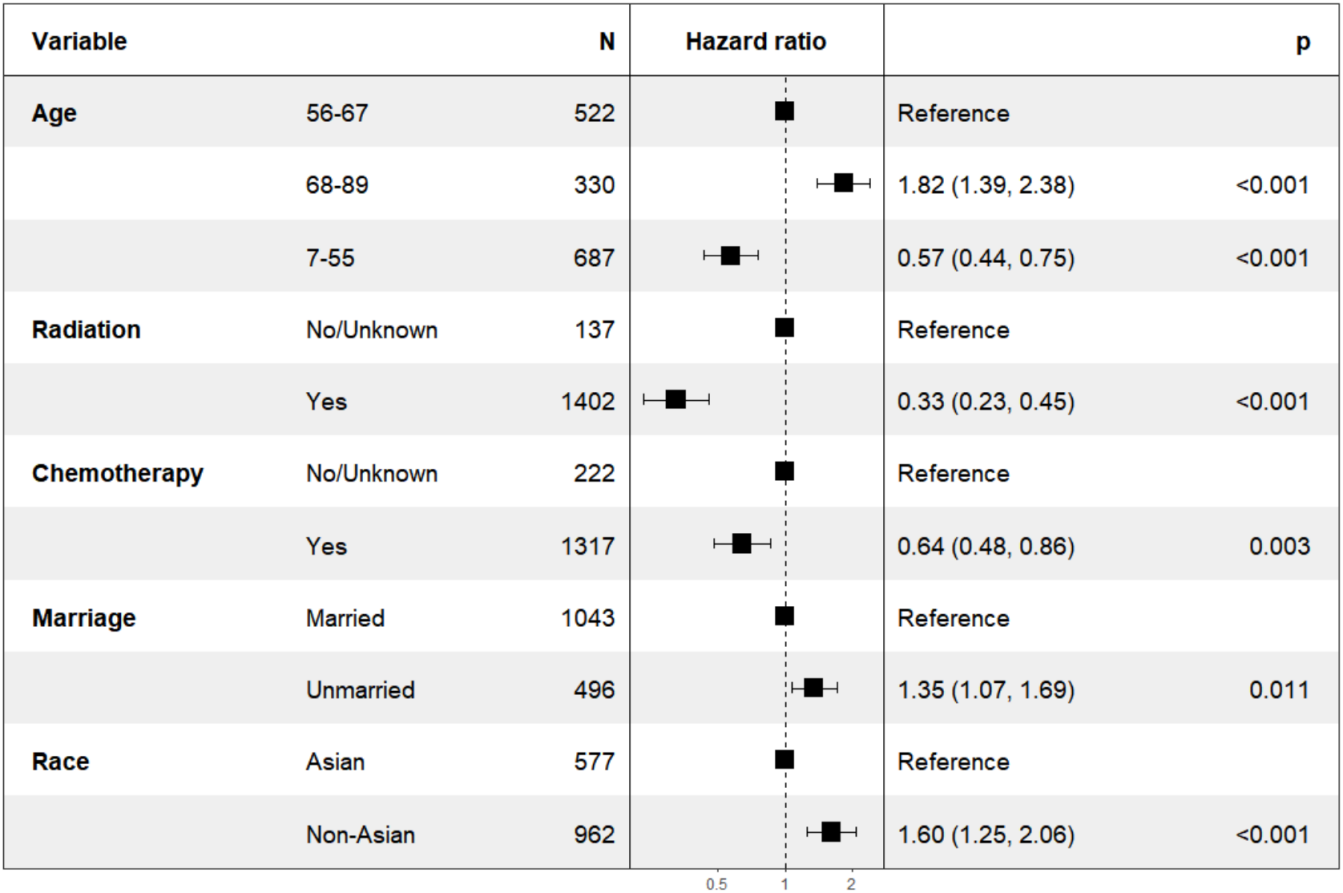
Forest map of patients with stage II NPC.

**Figure 2:**
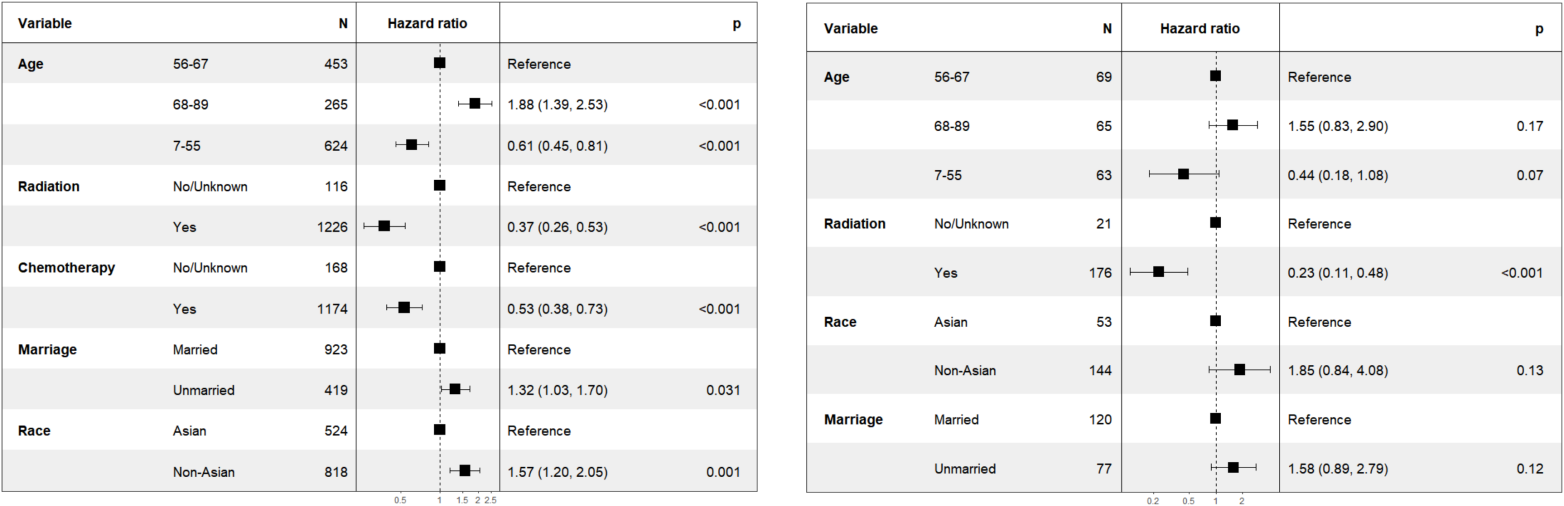
Forest map of patients with sub-stages NPC (T1-2N1M0 stage: left; T2N0M0 stage: right).

**Figure 3:**
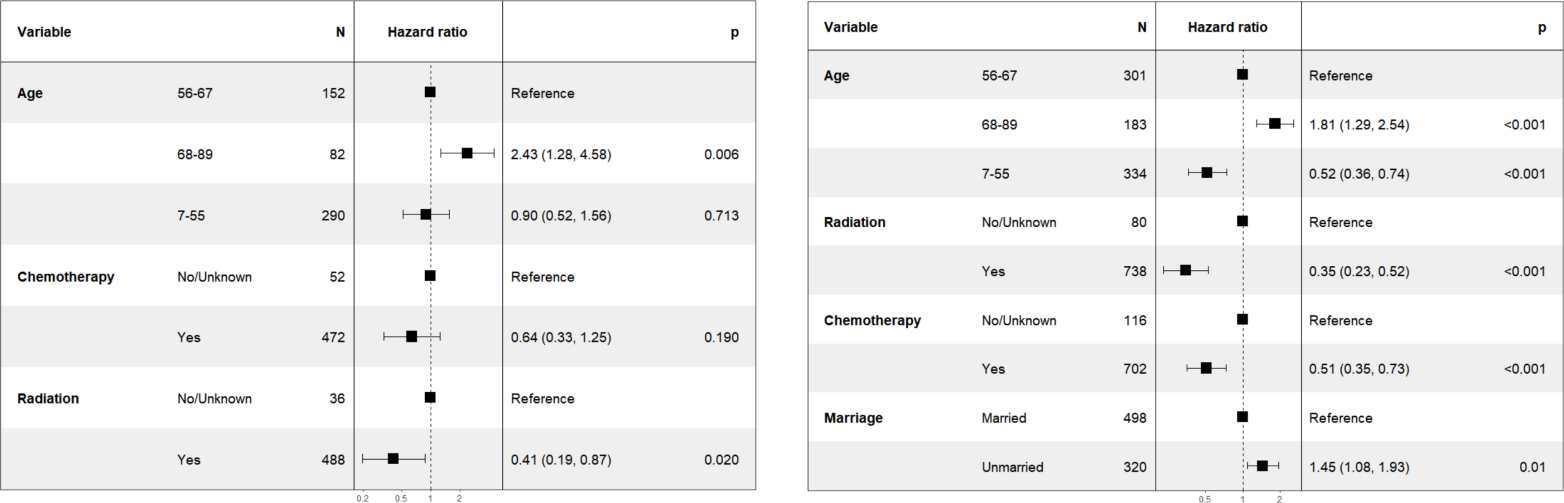
Forest map of Asian (left) and non-Asian (right) patients with T1-2N1M0 stage NPC.

**Figure 4:**
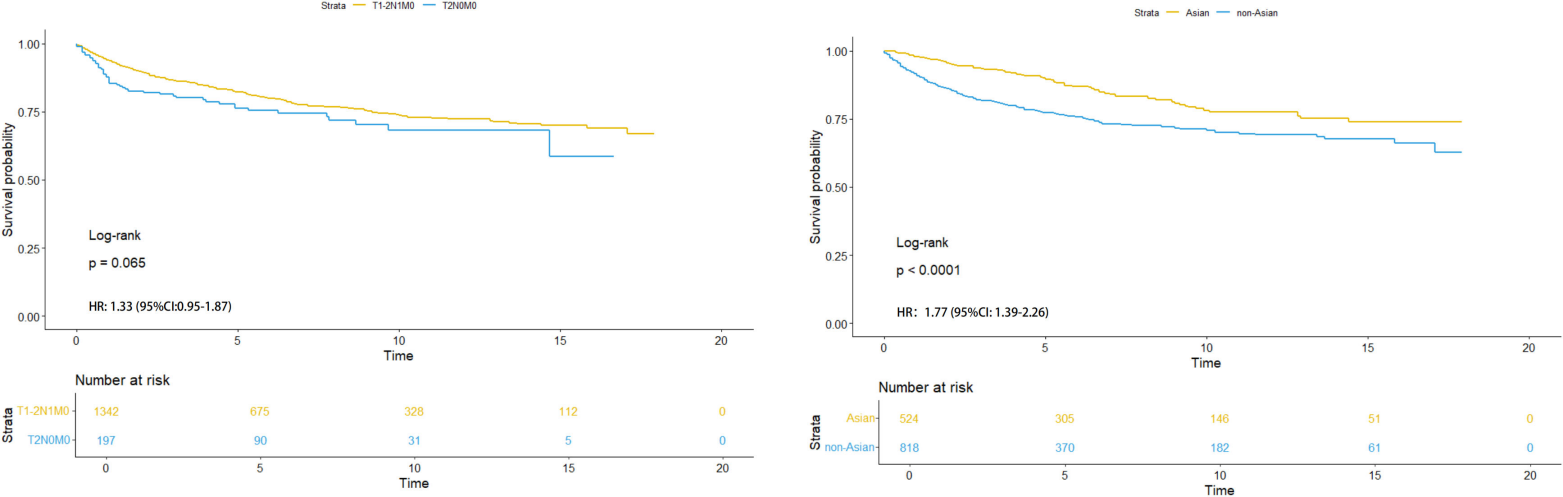
HR analysis of sub-stages (left) and different races (Asian and non-Asian) with T1-2N1M0 stage (right).

**Figure 5:**
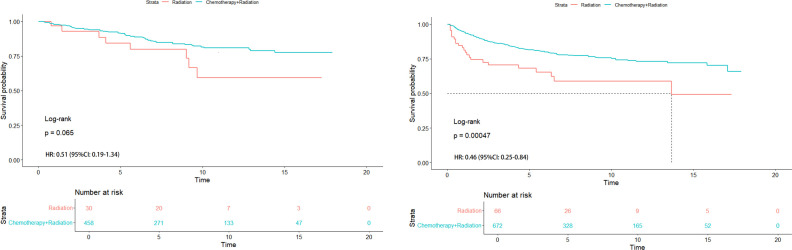
HR analysis of radiation alone vs chemoradiotherapy in Asian (left) and non-Asian (right) patients with T1-2N1M0 stage.

The regression analysis was conducted using Rstudio software (version: 2024.4.1.748) and R (version: 4.4.1). Data were extracted from the SEER database. Continuous variables were expressed as mean ± standard deviation, while counting variables were presented as numbers of cases and percentages. Multivariate regression analyses were performed using proportional hazards regression models, specifically the Cox model. The ‘MASS’ package was utilized to screen variables, employing the backward elimination approach. Reference variables were automatically generated by the ‘MASS’ package and were shown in **Figures 1**–**3**. The variables were in multivariate regression analyses after screening. Kaplan-Meier method was used to compare survival status between sub-stages and it was also used to compare the survival status between Asian and Non-Asian patients with T1-2N1M0 stage in **Figures 4** and **5**. It was generated using the ‘ggplot2’ package. Forest plots were created with the ‘forest model’ package to visualize the multivariate regression analysis. The age cut-off points were determined using X-tile software (version 3.6.1), resulting in the classification into three age groups: 7-55 years, 56-67 years, and 68-89 years.

### 2.5 Statistical process

R was also the statistical software. The log-rank test was used for statistical analysis, and statistical tests were 2-sides. Statistically significant for multivariate regression analysis and Kaplan-Meier analysis results were defined as *P* ≤ 0.05.

## 3 Result

### 3.1 Baseline characteristics of patients with stage II NPC

**Figure 6:**
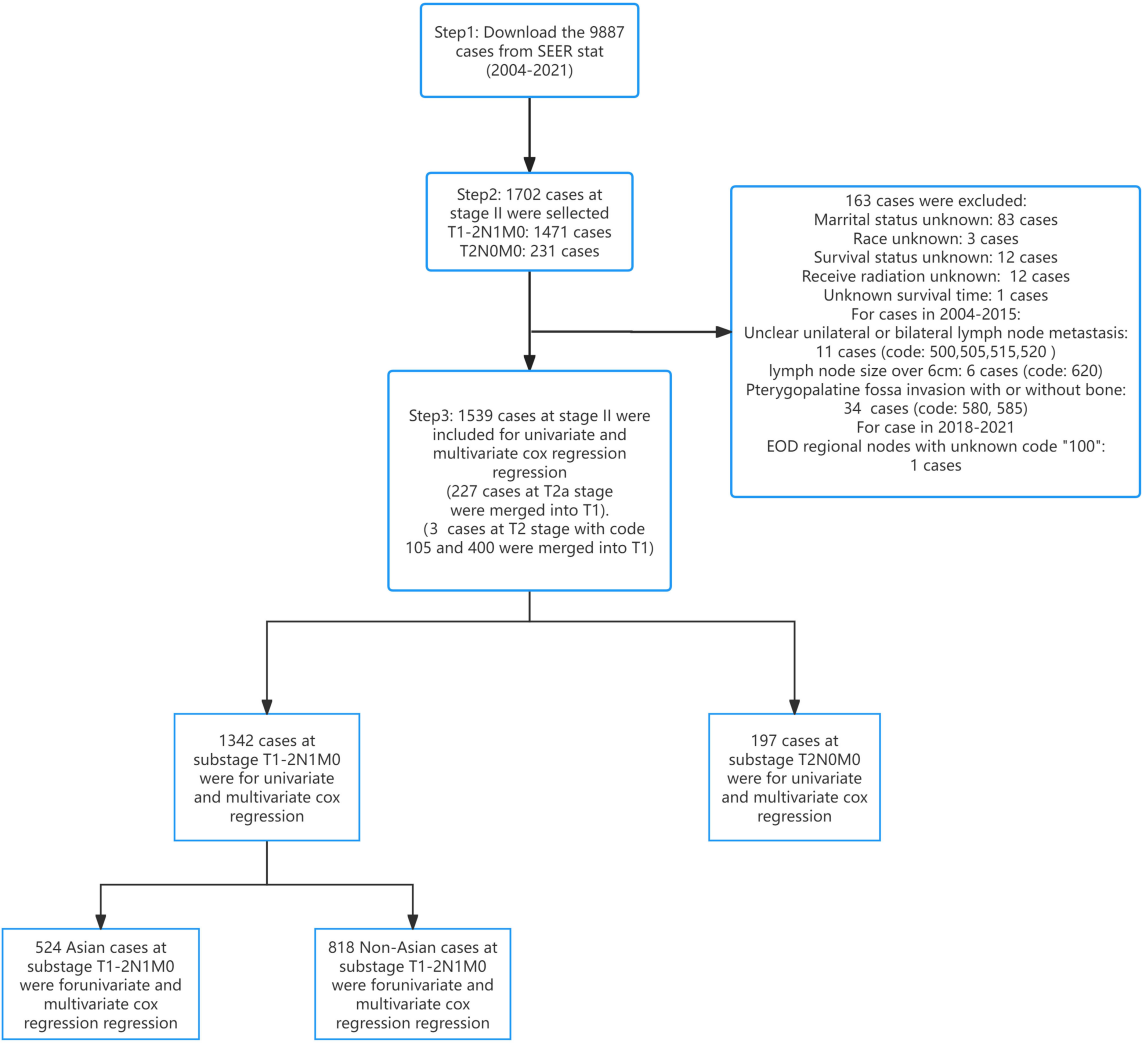
Flow chart of cases inclusion and exclusion.

**Figure 6** displayed the process of case inclusion and exclusion. Excluding the cases that were deleted due to incomplete information, we deleted 34 cases with pterygopalatine fossa invasion for T stage with code “580” and “585”. We excluded 11 cases where the location of cervical lymph node metastasis could not be determined as unilateral or bilateral for the N stage with code “500”, “505”, “515”, “520”. We also excluded 6 cases where the maximum diameter of the lymph nodes exceeded 6 cm with code “620”, and 1 case with code 100, which could not be interpreted due to unclear code information. We also performed conversions for cases that had staging issues: for the T stage, 227 cases originally classified as T2a were reclassified to T1, and cases coded as 105 (2 instances) and 400 (1 instance) were also reclassified to T1. This conversion further reduced the number of patients with T2 stage compared to T1 stage in our study.

**Table 2** showed that a total of 1,539 patients with stage II NPC were included in the study. Of these, 1,342 patients had a stage of T1-2N1M0, and only 197 patients had a stage of T2N0M0. The age distribution among the total patient population was as follows: 687 individuals (44.6%) were aged 7-55, 522 (33.9%) were aged 56-67, and 330 (21.5%) were aged 68-89. In terms of ethnicity, non-Asian patients were the most represented with 962 individuals (62.5%), followed by Asian patients with 577 (37.5%). Regarding gender, the disease prevalence was higher in males than in females, with 1,056 males (68.6%) and 483 females (31.4%). In terms of marital status, married individuals were more numerous than unmarried ones, with 1,043 married (67.8%) and 496 unmarried (32.2%). Overall, 1,317 individuals (85.6%) received chemotherapy, and 1,402 (91.1%) received radiation. 1271 (82.6%) patients received both radiation and chemotherapy. Only 46 (3.0%) patients received chemotherapy alone accounting for the smallest percentage. It also should be noted that 1130 (84.2%) patients with T1-2N1M0 stage received both radiation and chemotherapy. 141 (71.6%) patients with T2N0M0 stage received both radiation and chemotherapy.

**Table 2:** Baseline characteristic of patients with stage II NPC.

	All (n=1539)	T1-2N1M0 (n=1342)	T2N0M0 (n=197)
Age			
7-55	687 (44.6%)	624 (46.5%)	63 (32.0%)
56-67	522(33.9%)	453 (33.8%)	69 (35.0%)
68-89	330(21.5%)	265 (19.7%)	65 (33.0%)
Race			
Asian^c^	577 (37.5%)	524(39.0%)	53 (26.9%)
Non-Asian^d^	962 (62.5%)	818(61.0%)	144 (73.1%)
Sex			
Male	1056 (68.6%)	923 (68.6%)	120 (61.0%)
Female	483 (31.4%)	421 (31.4%)	77 (39.0%)
Marital status			
Married	1043 (67.8%)	923 (68.7%)	120 (61.0%)
Unmarried	496 (32.2%)	419(31.3%)	77 (39.0%)
Survival time			
Mean ± SD	6.15 ± 4.94	6.27 ± 5.02	5.31 ± 4.26
Status			
Alive or dead of other disease	1221 (79.3%)	1072 (79.9%)	149 (75.6%)
Dead attribute of this carcinoma	318 (20.7%)	270 (20.1%)	48 (24.4%)
T stage			
T1	981 (63.7%)	981 (73.1%)	NA
T2	558 (36.3%)	361 (26.9%)	197 (100%)
N stage			
N0	197 (12.8%)	NA	197 (100%)
N1	1342 (87.2%)	1342(100%)	NA
Chemotherapy			
Yes	1317 (85.6%)	1174 (87.5%)	143 (72.6%)
No	222 (14.4%)	168 (12.5%)	54 (27.4%)
Radiation			
Yes	1402 (91.7%)	1226 (91.4%)	176 (89.3%)
No	137 (8.9%)	116 (8.6%)	21 (10.7%)
Chemotherapy alone	46 (3.0%)	44 (3.2%)	2 (1.0%)
Radiation alone	131 (8.5%)	96 (7.2%)	35 (17.8%)
Both radiation and chemotherapy	1271 (82.6%)	1130 (84.2%)	141 (71.6%)
Neither radiation nor chemotherapy	91 (5.9%)	72 (5.4%)	19 (9.6%)

^c^Asian included: 284 Chinese, 115 Filipinos, 9 Cambodians, 71 Vietnamese, 4 Thais, 3 Hmong, 13 Koreans, 13 Japanese, 9 Laotians, and 56 classified under “Other Asian”, a category defined by SEER but not explicitly displayed.

^d^Non-Asian included: 745 White, 153 Black, and 64 “Other”. The “Other” category included Chamorro, American Indian, Alaska Native, Guamanian, Polynesian, Samoan, Tongan, Hawaiian, Asian Indian, Pakistani, and those generally described as “other” without a specific race mentioned.

### 3.2 Multivariate Cox regression analysis

In our analysis of patients with overall stage II, we employed the backward elimination method to select all variables from **Table 2**. The variables that remained after screening were: age, race, marriage, radiation, and chemotherapy. These variables were incorporated into multivariate Cox regression, all of which were statistically significant as depicted in **Figure 1**. The HR for chemotherapy was 0.64 (95% CI: 0.48-0.86, *P*=0.003). For radiation, the HR was 0.33 (95% CI: 0.23-0.45, *P*<0.001).

In the subgroup analysis for patients with T2N0M0 stage, as illustrated in **Figure 2** (right), the variable “chemotherapy” was eliminated by the backward elimination method. The remaining variables: age, race, radiation, and marriage—underwent further analysis, with only radiation demonstrating statistical significance. The HR for radiation was 0.23 (95% CI: 0.11-0.48, *P*<0.001). Race showed no significant difference with HR of 1.85 (95% CI: 0.84-4.08, *P*=0.13).

Furthermore, as shown in **Figure 2** (left), for patients classified with T1-2N1M0 stage, variables including age, race, chemotherapy, radiation, and marriage were retained by the backward elimination method in the multivariate analysis, and each was found to be statistically significant.

### 3.3 Multivariate Cox regression analysis of Asian and non-Asian patients with T1-2N1M0 stage

To further validate the results, It was hypothesized that the effect of chemotherapy might differ between Asian and non-Asian patients. We therefore performed a subgroup analysis for patients with T1-2N1M0 stage, categorizing them into Asian (n=524) and non-Asian (n=818) groups. The findings were presented in **Figure 3** (left). Within the Asian subgroup, chemotherapy showed no significant survival advantage, with a HR of 0.64 (95% CI: 0.33-1.25, *P*=0.19). Conversely, in the non-Asian subgroup, as illustrated in **Figure 3** (right), chemotherapy was linked to improve CSS, with a HR of 0.51 (95% CI: 0.35-0.73, *P*<0.001).

### 3.4 Kaplan-Meier method analysis

In **Figure 4** (left), we found that there was no significant difference in Kaplan-Meier analysis between T1-2N1M0 stage and T2N0M0 stage (*P*=0.065). The HR value is 1.33 (95% CI: 0.95-1.87). In **Figure 4** (right), it was also found that there was a significant difference between Asian and non-Asian patients with T1-2N1M0 stage (*P*<0.0001), and non-Asian patients were at high risk. The HR value was 1.77 (95% CI: 1.39-2.26).

In order to further understand the survival impact of chemotherapy on T1-2N1M0 stage NPC patients, we conducted a survival analysis comparing patients who received radiotherapy alone to those who received chemoradiotherapy. In **Figure 5** (left), No significant difference was found in Kaplan-Meier analysis in Asian patients with T1-2N1M0 stage (*P*=0.065). The HR value was 0.51 (95%CI: 0.19-1.34). However, Significant difference was found in Kaplan-Meier analysis in non-Asian patients with the same stage (*P*<0.001) in **Figure 5** (right). The HR value was0.46 (95%CI: 0.25-0.84).

## 4 Discussion

There were three controversies that needed clarification in this section: first, whether chemotherapy offered a survival advantage for patients with stage II NPC; second, what the effect of chemotherapy were on different sub-stages; and third, what the effect of chemotherapy were on Asian and non-Asian races. The discussion on these existing controversies was as follows:

### 4.1 Controversy 1: chemotherapy for stage II: yes or no?

Perspective 1: It was believed that chemotherapy could not improve the survival of patients with stage II NPC in most studies conducted over the past 10 years. As early as 2015, a review questioned whether chemotherapy was necessary for NPC patients with stage II (9). Later, two retrospective studies of patients with stage II NPC reported that chemotherapy did not confer survival benefits (10, 11) and One was involved with only CCRT. The other was involved with three types including neoadjuvant, adjuvant chemotherapy (AC), and CCRT. A retrospective analysis with 272 patients with stage II NPC also indicated that adding induction chemotherapy (IC) to CCRT did not improve the overall survival, besides increased treatment-associated adverse events (12). Another propensity score-matched analysis which included 450 NPC patients with stage II showed the same opinion (13). A retrospective study with up to 15 years of follow-up also noted that chemoradiotherapy did not confer additional survival benefits either (14). The types of chemotherapy included CCRT and IC. Two other meta-analyses also indicated that chemoradiotherapy (including CCRT) did not enhance the overall survival of patients with stage II (15, 16). As early as 2022, the findings from a multicenter randomized controlled trial involving 341 patients with stage II showed that the 3-year failure-free survival rate of IMRT alone was not inferior to that of CCRT. It also pointed out whether the result applicable for nonendemic populations was unknown (17) and it contained 45 patients with stage T3N0M0. Furthermore, a study of 289 patients with stage II and T3N0M0 stage revealed that adding chemotherapy to IMRT did not improve overall survival (18). The ESMO concurred with this view and suggested that IMRT was recommended for NPC patients with stage II (IIA evidence) in the guidelines (7).

Perspective 2: On the contrary, another view held that CCRT helped to improve the overall survival rate of patients with stage II NPC. For instance, a 10-year study analysis of 199 cases with stage II NPC reported that CCRT had survival benefits compared to radiotherapy alone (19), which could improve the 5-year survival rate. However, it should be noted that this research included 31 patients with N2 stage. A more recent study, with a sample size of 220, observed that CCRT improved the 5-year survival rate compared to IMRT in patients with stage II NPC who were older than 60 years of age (20). Our results suggested that chemotherapy could improve the CSS of NPC patients with stage II, which was contrary to Opinion 1. However, a definitive conclusion could not be drawn from these findings.

There were two reasons for the controversy: firstly, the trials or meta-analyses mentioned shared a common characteristic—the number of patients with T1-2N1M0 stage (ranging from 86 to 1,563 patients) was greater than the number of patients with T2N0M0 stage (ranging from 0 to 361 patients). In our study, the number of patients with T1-2N1M0 stage was also significantly higher than that of patients with T2N0M0 stage (1,342 patients *vs*. 197 patients). The effect sizes were more strongly influenced by the number of patients at the T1-2N1M0 stage. The impact of chemotherapy on the survival of patients with stage II NPC needed further clarification through subgroup analysis in our study. Secondly, all of the aforementioned trials were conducted in Asian countries, with the majority originating from China. Although Southeast Asian countries and China have a high incidence of NPC, these results should not be assumed to represent the effect of chemotherapy in other races worldwide. Our study included both Asian and non-Asian races, with 818 non-Asian patients. This diversity might have affected the effect size of chemotherapy. In summary, It was not rigorous to take stage II as a criterion for the use of chemotherapy, which was consistent with previous study (21). Therefore, there were other controversies regarding the two sub-stages.

### 4.2 Controversy 2: chemotherapy for T2N0M0 yes or no?

For T2N0M0 stage (except adverse features), Guidelines showed the same opinion (6, 7, 22): IMRT alone. We further performed subgroup analyses (T1-2N1M0, T2N0M0). It was found that chemotherapy had no survival benefit in patients with T2N0M0 stage. This was in line with the guidance opinions of ESMO and CSCO. A machine learning model based on MRI images of NPC patients also found that patients with T2N0M0 stage did not benefit from chemotherapy (23). In the latest suggestion of NPC TNM stage modification, it has been suggested that T2N0M0 be downgraded to AJCC Ia stage with the same risk level as T1N0M0, and T1-2N1M0 to be downgraded to Ib stage. But the two sub-stages showed no statistical difference when T1N0M0 stage was set as reference in risk stratification (24). Our results indicated that there was no statistical difference in the Kaplan-Meier analysis for the two sub-stages after treatment. Previous research findings also indicated that T1-2N1M0 and T2N0M0 stages were not identified as prognostic factors for overall survival regardless of treatment (25).

In short, based on the results and the suggestions of the guidelines, patients with T2N0M0 could benefit from IMRT alone and did not require chemotherapy. It showed no survival advantage in CSS between Asian race and non-Asian race in our result.

### 4.3 Controversy 3: chemotherapy for T1-2N1M0 yes or no?

Our results indicated that patients with T1-2N1M0 stage could benefit from chemotherapy in **Figure 2** (left), which was also suggested by CSCO. Only a review further pointed that CCRT could improve the 5 year overall survival in patients with stage II (26). It should be noted that this result was based on US population. But no subgroup analysis was performed for Asian and non-Asian patients with T1-2N1M0 stage. It was suggested that patients with T2N1M0 stage have a higher rate of distant metastasis, so chemotherapy was needed (27). Whereas, a retrospective study including 100 NPC patients with T2N1M0 stage pointed out that chemotherapy failed to treat sub clinical metastatic foci effectively (21). In a retrospective study of patients with T1-2N1M0 and T3-4N0-1M0 stages, adding IC to CCRT did not improve survival outcomes for these patients (28). Another retrospective study showed that IMRT had comparable treatment outcomes for NPC patients with T1-2N1M0 stage, regardless of the chemotherapy regimen used (IC+CCRT, CCRT, CCRT+AC) (29).

Our findings contradicted those of most of existing studies in patients with T1-2N1M0 stage, which suggested no significant difference in survival benefit between chemoradiotherapy and radiotherapy alone for patients with T1-2N1M0 stage (17, 20, 30, 31). Since these studies were based on Chinese populations, we conducted a subgroup analysis to distinguish between Asian and non-Asian patients with T1-2N1M0 stage NPC. We found that in the non-Asian population, chemotherapy significantly improved the CSS, with a HR of 0.51 (95% CI: 0.35-0.73, *P*<0.001) in **Figure 3** (right). In contrast, in the Asian population, there was no significant improvement in CSS for patients with T1-2N1M0 stage NPC, with a HR of 0.64 (95% CI: 0.33-1.25, *P*=0.190) in **Figure 3** (left). Our results concluded that there was no survival benefit from chemotherapy in Asian patients with NPC at T1-2N1M0 stage. It aligned with the ESMO guidelines. However, it was important to note that ESMO’s guideline were based on studies involving Asian populations without non-Asians. In conjunction with **Figures 3** and **4**, the ESMO recommendation for IMRT alone was deemed more suitable for Asian patients with stage II. The CSCO’s recommendation for CCRT was found to be more applicable to non-Asian patients with T1-2N1M0 stage and IMRT alone for patients with T2N0M0 stage regardless of race.

A previous study suggested that Asians seemed to have a disease-specific survival advantage (32) and race might influence the distribution of histology (33). Our results further indicated that chemotherapy was correlated with CSS in non-Asian patients with T1-2N1M0 stage NPC. As early as 2016, a retrospective study of 380 patients also found that non-Asians (101 patients) were more likely to benefit from CCRT compared with Asians (279 patients), although stage II was used as a reference and sub-stages were not distinguished (8). Our results also showed that non-Asians had a higher risk of survival in T1-2N1M0 stage in **Figure 4** (right).

Although the results did not show weak association between chemotherapy and CSS in non-Asian populations in this study, biases were still present. confounding factors existed. However, our findings suggested that chemotherapy should be as an option for non-Asian patients at T1-2N1M0 stage compared to previous studies. Our findings also indicated that region might not be a factor in this disease, but rather ethnicity. Because previous studies had primarily focused on Asian countries, with China being the most represented, whereas all of patients in this study were from the United States.

## 5 Conclusion

Chemotherapy is correlated with the cancer-specific survival in non-Asian patients with T1-2N1M0 stage nasopharyngeal carcinoma, but not in Asian patients at the same stage. For patients with the T2N0M0 stage, chemotherapy is not correlated with the cancer-specific survival rate, regardless of ethnicity.

## 6 Limitation

First, due to the limited information on radiotherapy, detailed data could not be obtained on specific radiation techniques, such as 2D-RT, IMRT. The dose and course of treatment were also unknown. These factors could cause bias. When chemotherapy itself was considered as a factor, the main source of bias was the uncertainty of the form of radiation, as there was clear evidence that IMRT was as effective as CCRT in patients with stage II (34). Chemoradiotherapy was better than 2d-RT alone (15, 35). Whether the effect size of 2d-RT or IMRT was dominant. Therefore, there were 2 possible reasons that caused bias of this paper:

1. The effect size of IMRT was dominant: our conclusion was consistent with the conclusions of the existing studies and further refined the existing findings for non-Asian patients with T1-2N1M0 stage, which concluded that there was no survival benefit in adding concurrent chemotherapy to IMRT (17, 36).
2. The effect size of 2d-RT was dominant: The conclusion would contradict to the results of previous studies. The possible result might be no racial difference in efficacy. Chemoradiotherapy would provide survival benefit for both Asian and non-Asian NPC patients with T1-2N1M0 stage and better than 2d-RT alone. Our results were at opposite.

To further understand the effect of chemotherapy on the survival of patients with NPC in T1-2N1M0 stage, we performed a survival analysis of patients treated with radiotherapy alone versus chemoradiotherapy. In Kaplan-Meier survival analysis, ethnic difference was present in patients with T1-2N1M0 stage. In **Figure 5**, we further pointed out that the differences between RT alone and chemoradiotherapy in Asian and non-Asian races still existed, which was consistent with the conclusion of this paper. This bias stemmed from the uncertainty of the effect size. Therefore, a randomized controlled trail regarding chemotherapy for non- Asian NPC patients with T1-2N1M0 stage will be needed in the future. In short our conclusion was more inclined to reason “(1)”.

Second, the most recent revision suggestion of the N stage classification identified that some patients classified as N1 exhibited G3 image-identified extranodal extension in imaging findings, suggesting an upgrade to N3 (24). It could not be excluded in our study, and it was unclear whether this factor exhibited racial tendencies.

Third, owing to the lack of information, we were unable to ascertain whether these NPC patients were associated with the Epstein-Barr virus or the human papilloma virus.

Fourth, the primary distinction between the seventh and eighth TNM classification of the N1 stage was the alteration of the regional boundary, shifting from the supraclavicular fossa to the cricoid cartilage. Consequently, the prognostic differences between the two editions post-treatment remained unknown due to this boundary change.

## Data Availability

The raw data supporting the conclusions of this article will be made available by the authors, without undue reservation.
